# Isokinetic Strength Recovery and Fear of Re-Injury After ACL Reconstruction in Male Soccer Players: A Retrospective Cohort Study

**DOI:** 10.3390/jcm15031243

**Published:** 2026-02-04

**Authors:** Matteo Interlandi, Luca Santini, Sebastiano Zuppardo, Franco Merlo, Giovanni Grazzini, Gilberto Martelli

**Affiliations:** 1 ”Performance” Rehabilitation and Medical Center, 53100 Siena, Italy; 2 ”Pegaso” Rehabilitation and Medical Center, 58100 Grosseto, Italy; 3 Easytech Research Department, 50032 Borgo San Lorenzo, Italy

**Keywords:** anterior cruciate ligament, isokinetic dynamometry, physical functional performance, fear, return to sport, soccer

## Abstract

**Background/Objectives**: Persistent strength deficits and psychological impairments may compromise return to sport (RTS) after anterior cruciate ligament reconstruction (ACLR). We investigate the relationship between thigh muscle isokinetic strength recovery at six months after ACLR and long-term psychological outcomes related to RTS in competitive male soccer players. **Methods**: Sixty male soccer players who underwent primary ACLR with bone–patellar tendon–bone autograft were retrospectively analyzed. Isokinetic testing of quadriceps and hamstrings was performed one week before surgery and six months post-surgery at 90°/s and 180°/s. Limb symmetry index (LSI) was calculated both pre- and post-operatively. At long-term follow-up (mean ≈ 4 years after RTS), athletes completed questionnaires assessing RTS status, ACL re-injuries, sport-related perceptions, and kinesiophobia using the Tampa Scale for Kinesiophobia (TSK). Statistical analyses were conducted to explore associations between post-operative LSI and TSK scores and to compare psychological and neuromuscular outcomes between athletes with and without ACL re-injury. **Results**: Absolute quadriceps and hamstring peak torque values significantly increased from pre- to post-surgery, with quadriceps strength deficits persisting only in the operated limb. However, quadriceps LSI significantly decreased post-operatively, while hamstring LSI remained stable. Pearson correlation analysis revealed a weak positive association between post-operative quadriceps LSI at 90°/s and TSK scores (r = 0.34). Overall, RTS rate was 91.7%, but a second ACL injury occurred in 18.2% of athletes. No significant differences were observed between re-injured and non-re-injured athletes in TSK scores or post-operative LSI values at either angular velocity (all *p* > 0.29). High kinesiophobia (TSK ≥ 37) was present in 56.7% of the cohort at long-term follow-up. **Conclusions**: Despite significant strength gains, quadriceps limb symmetry worsened six months after ACLR, with deficits confined to the operated limb, suggesting persistent neuromuscular inhibition. These physical deficits coexist with long-term kinesiophobia despite high RTS rates. The weak associations between strength symmetry and psychological outcomes highlight the multifactorial nature of RTS and support the need for an integrated physical, psychological, and neuro-cognitive approach to rehabilitation and RTS decision-making.

## 1. Introduction

Despite continuous advances in surgical techniques and rehabilitation protocols, a substantial proportion of athletes do not achieve full functional recovery following anterior cruciate ligament reconstruction [1]. In particular, persistent deficits in quadriceps and hamstring strength are frequently reported at six months after surgery, a time point commonly adopted in clinical practice to guide return-to-sport (RTS) decision-making [1,2]. Isokinetic testing represents one of the most reliable and objective methods for the assessment of knee extensor and flexor strength after ACLR, allowing for the standardized quantification of peak torque deficits and inter-limb asymmetries [1,2]. Previous studies have consistently shown residual inter-limb asymmetries, especially in quadriceps strength and activation, even when athletes meet traditional time-based RTS criteria [1,2,3,4]. Deficits in neuromuscular control may result in altered lower-limb biomechanics, including increased knee valgus and internal rotation moments, which reduce dynamic joint stability and increase ligament loading during cutting, pivoting, and landing tasks [3].

In parallel with physical impairments, psychological factors have emerged as critical determinants of RTS outcomes. Fear of re-injury, loss of confidence, and kinesiophobia are commonly reported after ACLR and may persist long after the athlete has returned to competition [5]. Importantly, psychological readiness does not necessarily develop simultaneously with physical recovery [5,6], and athletes who demonstrate acceptable physical function may still experience substantial psychological barriers to full performance. Although psychosocial interventions have been proposed as potential strategies to reduce kinesiophobia and improve RTS readiness, higher-quality evidence is still required to define their effectiveness and optimal timing within rehabilitation [7]. RTS decisions are still frequently based on isolated physical parameters, with limited systematic integration of psychological assessment into clinical decision-making models [6,7,8]. According to the current literature, readiness for return to sport after ACL reconstruction is determined by the restoration of muscle strength, knee stability, and neuromuscular control during functional tasks, rather than by time since surgery alone [9].

While strength recovery and psychological factors have been investigated separately, fewer studies have explored their long-term interaction. In particular, the relationship between early post-operative isokinetic strength recovery, evolution of limb symmetry, and long-term psychological outcomes remains poorly understood.

Six months after ACL reconstruction represents a critical milestone in the rehabilitation process, as it is frequently used in clinical practice as a reference point for clearance to progressive sport participation. However, this time-based milestone does not necessarily reflect complete neuromuscular recovery. Several authors have questioned the validity of using fixed temporal thresholds, highlighting that strength restoration, motor control, and inter-limb symmetry may follow different recovery trajectories [10].

In particular, improvements in absolute muscle strength may mask persistent deficits in voluntary activation and limb symmetry, especially at the quadriceps level. LSI is widely adopted as a clinical benchmark, yet emerging evidence suggests that LSI values may be influenced not only by recovery of the operated limb but also by compensatory strength gains in the contralateral limb. This phenomenon may lead to an apparent paradox whereby athletes demonstrate higher absolute strength but poorer symmetry, potentially increasing biomechanical and neuromuscular risk during high-demand sport tasks.

In parallel, psychological recovery appears to evolve independently from physical restoration [10]. Fear of re-injury and kinesiophobia may persist even after successful return to sport, suggesting that psychological readiness does not automatically accompany physical clearance [11]. Despite increasing recognition of these factors, the relationship between early post-operative neuromuscular recovery, particularly isokinetic strength and limb symmetry at six months, and long-term psychological outcomes remains insufficiently explored, particularly in soccer players exposed to high levels of cutting, pivoting, and contact demands.

Therefore, the primary aim of this retrospective cohort study was to investigate the association between thigh muscle isokinetic strength recovery and limb symmetry at six months after ACLR and long-term psychological outcomes related to RTS in competitive male soccer players. Secondary aims were to describe RTS rates, ACL re-injury incidence, limb symmetry indices (LSI) before and after surgery, and kinesiophobia at a mean follow-up of approximately four years after surgery.

Based on existing evidence, we hypothesized that residual quadriceps limb symmetry at six months after ACLR would be associated with higher levels of long-term kinesiophobia, and athletes sustaining a subsequent ACL injury would demonstrate different psychological and neuromuscular profiles compared with those without re-injury.

## 2. Materials and Methods

### 2.1. Study Design

This study was designed as a retrospective cohort study. Isokinetic strength data were collected during standard clinical practice in a sports rehabilitation setting as part of routine clinical assessments between 2020 and 2024 were retrospectively analyzed. Psychological outcomes and RTS-related data were extracted through questionnaires administered in 2025. The retrospective design was chosen to analyze isokinetic strength data routinely collected. This approach allowed for the investigation of long-term psychological outcomes in relation to early post-operative neuromuscular recovery without interfering with clinical decision-making processes or altering rehabilitation protocols.

### 2.2. Participants

Sixty male soccer players were included in the study (mean age at surgery 21.6 ± 4.5 years; body mass 74.65 ± 9.68 kg; height 1.79 ± 0.06 m). Athletes competed at recreational, competitive, and professional levels. Inclusion criteria were (1) primary unilateral ACL reconstruction, (2) participation in organized soccer prior to injury, (3) completion of a standardized post-operative rehabilitation program, and (4) availability of both pre- and post-operative isokinetic testing data. Exclusion criteria included previous ACL injury on either limb, multi-ligament knee injuries and the presence of neurological or musculoskeletal disorders potentially affecting lower limb function.

The mean time interval between ACL injury and surgical reconstruction was 8.18 ± 11.73 months.

### 2.3. Surgical Procedure

All anterior cruciate ligament reconstructions were performed by the same orthopedic surgeon using a bone–patellar tendon–bone (BPTB) autograft. Surgical technique and fixation methods were standardized across all procedures. The use of a single surgeon and a uniform graft choice was intended to minimize procedural variability and to limit potential confounding effects related to different surgical techniques, graft types, or fixation strategies on post-operative neuromuscular and strength recovery.

### 2.4. Rehabilitation Program

All athletes followed an identical post-operative rehabilitation program supervised by experienced sports physiotherapists. Rehabilitation was structured according to a phased and criterion-based progression while maintaining standardized time frames across participants. During the early rehabilitation phase, the primary goals were pain and effusion control, restoration of knee range of motion, and early quadriceps activation, with particular attention to minimizing arthrogenic muscle inhibition. Low-load isometric exercises, neuromuscular electrical stimulation when clinically indicated, and voluntary activation strategies were implemented to promote quadriceps engagement. The intermediate phase focused on progressive resistance training, with gradual increases in external load and training volume. Both bilateral and unilateral exercises were prescribed, including closed- and open-kinetic-chain movements. Controlled knee extension exercises were introduced within safe ranges of motion to progressively load the extensor mechanism while minimizing anterior tibial shear forces. Neuromuscular training emphasized balance, proprioception, and movement quality to enhance lower-limb control during functional tasks. The late rehabilitation phase incorporated advanced strength training, plyometric exercises, and sport-specific drills. Athletes were progressively re-exposed to running, cutting, jumping, and change-of-direction tasks, with increasing intensity and complexity. Progression through rehabilitation phases was based on predefined clinical criteria, including pain-free execution, adequate range of motion, and tolerance to increasing neuromuscular and mechanical demands.

### 2.5. Isokinetic Strength Assessment

Isokinetic testing was performed using a Genu 3 isokinetic dynamometer (Easytech, Florence, Italy). Assessments were conducted one week prior to surgery and at six months post-operatively. The six-month post-operative time point was selected, as it is commonly used in clinical practice to guide return-to-sport decision-making. Concentric quadriceps (Q) and hamstrings (H) strength were evaluated bilaterally at angular velocities of 90°/s and 180°/s. At 90°/s, four maximal repetitions were performed to assess peak torque production, whereas at 180°/s, twenty maximal repetitions were completed to evaluate the ability to sustain muscle performance during repeated high-velocity contractions. Peak torque (PT) values were recorded for each muscle group and limb. Limb symmetry index (LSI) was calculated as the ratio between operated and non-operated limb PT values, expressed as a percentage. Additional individual isokinetic strength values and limb symmetry index data are reported in Table S1.

### 2.6. Psychological Outcomes and Re-Injury

At a mean follow-up of approximately four years after RTS, athletes completed a questionnaire investigating RTS status, ACL re-injuries, and subjective perceptions during sport activity. Re-injury was defined as a subsequent ipsilateral or contralateral ACL rupture occurring after RTS. In addition, kinesiophobia was assessed using the Tampa Scale of Kinesiophobia (TSK): a cut-off value of 37 was used to distinguish between low and high kinesiophobia, in accordance with the previous literature [5]. Additional individual psychological outcomes, including Tampa Scale of Kinesiophobia and return-to-sport-related data, are reported in Supplementary Table S2.

### 2.7. Statistical Analysis

Descriptive statistics were calculated for all variables and reported as mean ± standard deviation (SD) or percentages. Paired Student’s *t*-tests were used to assess pre- to post-operative changes in peak torques and limb symmetry index values. Associations between post-operative LSI values at six months and long-term TSK scores were assessed using Pearson correlation coefficients. To evaluate differences in psychological and physical outcomes between athletes who sustained a second ACL injury and those who did not, Mann–Whitney U tests were performed for TSK scores and post-operative LSI values (quadriceps and hamstring at 90°/s and 180°/s). Effect sizes (r) were calculated. For non-parametric comparisons, the U statistic was calculated and standardized to obtain the Z value, and effect size was estimated using the r coefficient (r = |Z|/√N),whereNrepresentsthetotalsamplesize. Statistical significance was set at *p* < 0.05. All statistical analyses were performed using standard statistical software (Microsoft Excel, version 16.101.3, Microsoft Corp., Redmond, WA, USA). Detailed individual data and complete statistical outputs supporting the isokinetic and psychological analyses are reported in Tables S1 and S2.

## 3. Results

### 3.1. Return to Sport and Re-Injury

**Figure 1:**
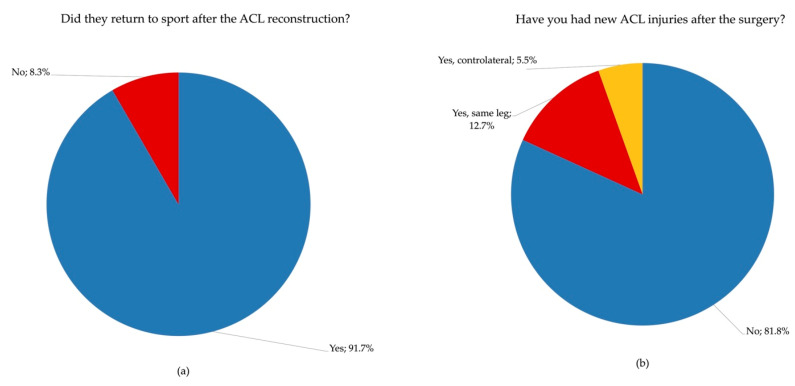
Return-to-sport rate (**a**) and ACL re-injury distribution (no re-injury; contralateral ACL injury; ipsilateral ACL injury) (**b**).

At long-term follow-up, 91.7% of athletes returned to sport, whereas the remaining participants did not resume sport participation (Figure 1a). Among athletes who successfully returned to sport, 81.8% did not sustain any further ACL injuries, while a second ACL injury was reported in 18.2% of cases during the follow-up period. (Figure 1b). The mean time to return to sport was 12.45 ± 6.22 months after surgery.

**Figure 2:**
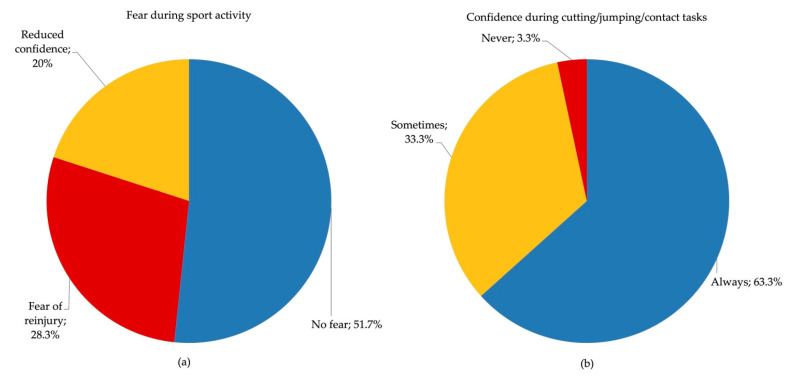
RTS perception outcomes: fear during sport activity (**a**) and confidence during cutting/jumping/contact tasks (**b**).

Regarding psychological perceptions during sport participation, approximately half of the athletes reported no fear, whereas the remaining participants perceived fear of re-injury or reduced confidence in the operated knee (Figure 2a). Furthermore, during soccer-specific activities requiring change in direction, jumping, or physical contact, approximately two-thirds of the athletes reported full confidence, while the others reported occasional insecurity and persistent lack of confidence during performance (Figure 2b).

### 3.2. Kinesiophobia

**Figure 3:**
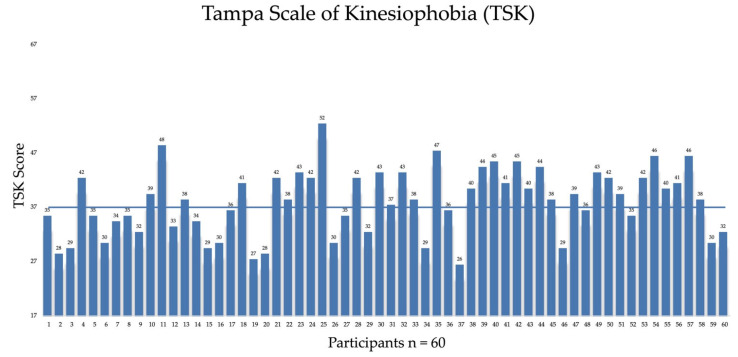
Individual Tampa Scale for Kinesiophobia (TSK) scores for the study sample (*n* = 60) at long-term follow-up after return to sport. The horizontal line indicates the kinesiophobia cut-off value (TSK = 37).

Tampa Scale of Kinesiophobia (TSK) scores ranged from 26 to 52 with a mean value of 37.5 ± 6.1. Based on the established cut-off value, 56.7% of athletes were classified as having high kinesiophobia, whereas 43.4% were categorized as having low kinesiophobia (Figure 3). No significant difference in TSK scores was observed between athletes who sustained a second ACL injury and those who did not (Mann–Whitney U = 197.5; Z = −1.04; *p* = 0.30; r = 0.13). Individual TSK scores for all participants are reported in Table S2.

### 3.3. Peak Torque Changes

**Figure 4:**
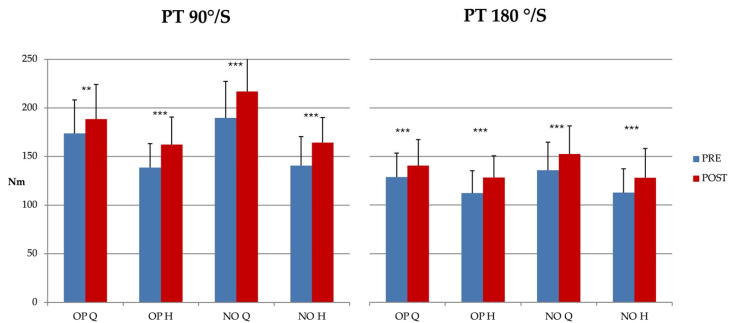
Knee isokinetic peak torques (PT, mean ± SD) assessed one week before (PRE) and six months after (POST) ACL reconstruction during two concentric isokinetic tests performed at different angular velocities: 90°/s and 180°/s. Measurements were obtained for quadriceps (Q) and hamstrings (H) in the operated (OP) and not-operated (NO) limbs. PRE vs. POST statistical comparison: ** = *p* < 0.01; *** = *p* < 0.001.

Absolute quadriceps and hamstring peak torque (PT) values significantly increased from pre-operative to post-operative assessments both for quadriceps and hamstring PT values at the two angular velocities (Figure 4). However, when comparing limbs at the post-surgery assessment, quadriceps PT values remained significantly lower in the operated limb compared with the non-operated one at both angular velocities (*p* < 0.01).

### 3.4. Limb Symmetry Index

In the comparison between pre- and post-surgery strength values, despite the significant improvements in absolute quadriceps strength, limb symmetry index (LSI) analysis revealed an opposite trend. Specifically, at six months post-surgery, quadriceps LSI significantly decreased compared with pre-operative values, whereas hamstring LSI remained stable over time (Table 1). Individual pre- and post-operative limb symmetry index values are provided in Table S1.

### 3.5. Association Between Limb Symmetry and Kinesiophobia

Correlation analyses were performed to investigate the association between post-operative limb symmetry indices (LSI) assessed at six months after ACL reconstruction and long-term kinesiophobia measured using the Tampa Scale for Kinesiophobia (TSK). A weak positive correlation was observed between post-operative LSI quadriceps assessed at 90°/s at six months after surgery and TSK scores (r = 0.34), indicating that greater quadriceps limb symmetry at low angular velocity was modestly associated with higher levels of kinesiophobia at long-term follow-up. In contrast, correlations between TSK scores and hamstring LSI at 90°/s were negligible (r = 0.09). Similarly, no meaningful associations were observed between TSK scores and quadriceps or hamstring LSI assessed at 180°/s (quadriceps: r = 0.05; hamstrings: r = −0.09).

### 3.6. Association Between Kinesiophobia and Re-Injury

No significant differences in long-term Tampa Scale of Kinesiophobia (TSK) scores were observed between athletes who sustained a second ACL injury and those who did not (Mann–Whitney U = 197.5, Z = −1.04, *p* = 0.30). The magnitude of the difference was small, as indicated by a small effect size (r = 0.13).

### 3.7. Association Between Limb Symmetry and Re-Injury

No significant differences in post-operative LSI values were observed between athletes who sustained a second ACL injury and those who did not, for either muscle group or angular velocity (Table 2).

## 4. Discussion

The main finding of this study is that competitive male soccer players exhibit persistent quadriceps inter-limb asymmetry six months after ACL reconstruction, despite significant improvements in absolute strength, high return-to-sport (RTS) rates, and near-complete hamstring recovery. This dissociation between absolute strength gains and limb symmetry highlights a critical limitation of strength-only and time-based RTS criteria [1,2]. From a clinical perspective, this dissociation may contribute to a false perception of recovery, whereby improvements in absolute strength lead to premature progression through rehabilitation phases or return-to-sport clearance despite persistent neuromuscular asymmetries. Such discrepancies may not be detected when decision-making relies exclusively on isolated strength values or time-based milestones.

Isokinetic testing demonstrated that quadriceps strength increased from pre- to post-operative assessments in both limbs. However, post-surgery between-limb comparisons revealed that quadriceps strength remained consistently lower in the operated limb compared to the contralateral limb, indicating that the strength recovery was incomplete exclusively on the reconstructed side, as shown by several previous papers [1,2,3,4]. In parallel, the non-operated limb showed a greater absolute increase in quadriceps strength from pre- to post-operative testing, further contributing to the observed inter-limb asymmetry. These findings indicate that, although overall force-generating capacity improves, the operated limb fails to recover proportionally relative to the uninvolved limb [3,4].

Quadriceps asymmetry was more pronounced at lower angular velocity, suggesting impaired maximal force production rather than purely velocity-dependent deficits, a pattern widely reported following ACL injury and reconstruction [4]. This presentation is consistent with arthrogenic muscle inhibition (AMI), a condition characterized by persistent reductions in voluntary quadriceps activation following knee injury [8,9,10,11,12,13,14]. In addition, AMI is not solely driven by peripheral joint factors but is strongly influenced by spinal and supra-spinal adaptations, including altered afferent input, reflex inhibition, and reduced cortico-spinal excitability [13].

Neuro-physiological studies have demonstrated that ACL injury and reconstruction are associated with changes in motor cortex organization, cortico-spinal drive, and sensorimotor integration, which may persist despite intensive rehabilitation and apparent strength recovery [15]. From this broader brain–knee perspective, ACL injury should be considered a neuro-muscular and neuro-cognitive condition, rather than a purely mechanical lesion [15,16]. Contemporary conceptual frameworks emphasize the role of neuro-cognitive and neuro-physiological dysfunctions in ACL injury and advocate for the integration of neuro-cognitive strategies within rehabilitation and RTS testing protocols [16]. The persistence of kinesiophobia several years after RTS further supports this interpretation [5,17]. These neurophysiological alterations may have relevant functional consequences during sport-specific tasks that require rapid decision-making, anticipatory motor control, and efficient integration of sensory information. Altered central motor processing may therefore contribute not only to residual strength asymmetries but also to impaired movement confidence and increased perception of threat during high-demand actions.

In the present cohort, more than half of the athletes demonstrated high kinesiophobia at long-term follow-up, in line with systematic reviews evidence showing that fear of re-injury is highly prevalent after primary ACL reconstruction and influenced by multiple interacting physical and psychological factors [5,12,18,19]. Notably, this psychological burden persisted despite a high overall RTS rate, revealing a paradox whereby returning to sport does not necessarily equate to psychological readiness [6,20]. Fear of re-injury and reduced confidence were particularly evident during high-demand tasks such as cutting, jumping, and physical contact, activities that place substantial demands on neuromuscular control and rapid force generation [18,19,20]. These findings suggest that return-to-sport clearance should not be interpreted as a binary outcome but rather as a continuum, in which physical, psychological, and neuro-cognitive readiness may evolve at different rates. The persistence of fear-related symptoms despite successful RTS highlights the need for ongoing monitoring beyond the initial return to competition.

Importantly, the present analyses indicate that long-term kinesiophobia was not strongly explained by either neuromuscular symmetry or re-injury occurrence [9]. Correlation analyses revealed only a weak positive association between quadriceps limb symmetry index assessed at 90°/s six months after surgery and long-term TSK scores, while associations involving hamstring symmetry or higher testing velocity were negligible. These findings suggest that early post-operative limb symmetry accounts for only a small proportion of the variance in long-term psychological outcomes.

Similarly, Mann–Whitney analyses demonstrated no significant differences in TSK scores between athletes who sustained a second ACL injury and those who did not, indicating that re-injury status alone does not appear to be a dominant determinant of persistent kinesiophobia. In parallel, no significant differences in quadriceps or hamstring LSI at either 90°/s or 180°/s were observed between re-injured and non-re-injured athletes, with small effect sizes across all comparisons. Collectively, these results suggest that psychological feat and neuromuscular asymmetry may follow partially independent recovery [9,10].

Residual quadriceps asymmetry and central motor control alterations may increase threat perception during these tasks, reinforcing fear-avoidance behaviors and potentially contributing to maladaptive movement strategies [15,16]. This observation is clinically relevant, as previous papers showed that failure to meet objective RTS discharge criteria is associated with a markedly increased risk of ACL graft rupture [18,20,21]. However, the absence of significant differences in neuromuscular parameters between re-injured and non-re-injured athletes in the present cohort highlights the limitations of relying on isolated physical metrics to explain complex RTS outcomes. Moreover, substantial heterogeneity exists in RTS clearance criteria across clinical practice, often relying on time-based thresholds rather than objective neuromuscular benchmarks [10,22].

Although quadriceps LSI values around 90% are often considered acceptable, emerging evidence suggests that residual strength and activation deficits may persist even when these thresholds are met [4,8]. Persistent quadriceps weakness following ACLR has been consistently documented and is associated with altered movement strategies and increased joint loading [3,4,20]. In addition, cohort-based decision rules using objective discharge criteria have been proposed to reduce re-injury risk, reinforcing the need to move beyond isolated measures [22].

Future studies should adopt prospective and longitudinal designs to better elucidate the temporal relationships between early neuromuscular recovery, central motor adaptations, and long-term psychological outcomes. The integration of objective neuromuscular assessments with psychological screening and neuro-cognitive testing may provide a more comprehensive framework for return-to-sport decision-making.

Taken together, these findings challenge the adequacy of RTS decision-making models based solely on time from surgery or isolated strength thresholds [20]. A multidimensional RTS framework, integrating isokinetic strength, limb symmetry, psychological readiness, and neuro-cognitive function appears essential to optimize long-term outcomes and reduce re-injury risk [7,16,17].

### 4.1. Limitations

This study has several limitations that should be acknowledged. First, the retrospective design limits causal inference and precludes control over rehabilitation exposure, training load, and external sport participation throughout the recovery process. Although all athletes followed a standardized rehabilitation protocol, individual differences in adherence, competitive demands, and off-field training activities could not be fully controlled and may have influenced neuromuscular and psychological outcomes.

Second, direct measures of neuromuscular activation, cortical excitability, or neuro-cognitive function were not available, limiting mechanistic interpretation of the observed strength asymmetries and psychological responses.

Third, the time interval between ACL injury and surgical reconstruction was relatively long in some athletes. Although this reflects real-world clinical timelines, prolonged pre-operative periods may influence neuromuscular status at the time of surgery and could have contributed to inter-individual variability in early post-operative strength recovery.

Fourth, psychological outcomes were assessed only at long-term follow-up using self-reported questionnaires, which may be subject to recall bias. The absence of repeated psychological assessments across different rehabilitation stages prevents an evaluation of the temporal evolution of kinesiophobia and confidence from early recovery to return to sport.

Finally, the inclusion of athletes competing at different performance levels may have influenced psychological responses and return-to-sport expectations, potentially contributing to variability in long-term outcomes.

### 4.2. Clinical Implications

The present findings have important clinical implications for rehabilitation planning and return-to-sport (RTS) decision-making after ACL reconstruction. Although isokinetic testing remains a cornerstone for objective strength assessment, the persistence of quadriceps inter-limb asymmetry despite significant absolute strength gains highlights that isokinetic strength alone is necessary but not sufficient to guide RTS clearance [1,2]. Reliance on isolated peak torque values or time-based criteria may lead to a premature return to sport, potentially exposing athletes to elevated neuromuscular and biomechanical risk [15,21].

The integration of limb symmetry analysis into routine clinical practice appears essential to identify residual deficits that may not be evident when considering absolute strength values alone [3,7]. However, the weak associations observed between early post-operative limb symmetry and long-term kinesiophobia suggest that neuromuscular recovery and psychological readiness may not evolve in parallel. This finding highlights the need for caution when interpreting strength symmetry as an indicator of psychological recovery.

In addition, the high prevalence of kinesiophobia observed at long-term follow-up highlights the importance of systematically assessing psychological readiness as part of RTS decision-making [4,5]. Addressing fear of re-injury and confidence deficits may require targeted interventions, including psychologically informed rehabilitation strategies, graded exposure to sport-specific tasks, and education aimed at reducing threat perception during high-demand actions [6,15,21].

Furthermore, the association between persistent quadriceps asymmetry, altered central motor control, and psychological factors suggests that rehabilitation should extend beyond peripheral strength restoration [12,13,14,15]. Interventions targeting arthrogenic muscle inhibition, neuromuscular coordination, and neuro-cognitive function may be necessary to optimize both physical and psychological recovery [7,12,15]. Taken together, these findings support the adoption of a multidimensional approach that integrates strength, symmetry, psychological readiness, and neuro-cognitive function to improve long-term outcomes and reduce the risk of secondary ACL injury [6,15,16].

### 4.3. Practical Applications

From a practical perspective, clinicians should interpret RTS clearance as a dynamic and progressive process rather than a binary outcome. Objective neuromuscular testing should be combined with psychological screening and task-specific exposure to guide individualized rehabilitation progression. Given the weak association observed between early post-operative limb symmetry and long-term kinesiophobia, clinicians should avoid relying on isolated physical metrics as direct measures for psychological readiness [10]. Monitoring both neuromuscular and psychological domains beyond the initial return to competition may help identify the athletes at risk of maladaptive movement strategies, feat-avoidance behaviors, or incomplete confidence restoration, thereby enabling timely, targeted interventions.

## 5. Conclusions

Male soccer players demonstrate persistent quadriceps neuromuscular asymmetry six months after anterior cruciate ligament reconstruction, despite significant improvements in absolute muscle strength, high return-to-sport rates, and near-complete hamstring recovery. Limb symmetry worsened from pre- to post-operative assessments, indicating incomplete recovery of the operated limb at a time point commonly used for return-to-sport decision-making.

At long-term follow-up, a substantial proportion of athletes reported kinesiophobia and fear of re-injury despite successful return to sport. However, only weak or negligible associations were observed between early post-operative limb symmetry and long-term psychological outcomes, and no significant differences in neuromuscular or psychological measures were found between re-injured and non-re-injured athletes.

Taken together, these findings suggest that physical and psychological recovery may follow partially independent trajectories after ACL reconstruction. Return-to-sport decision-making should therefore not rely exclusively on time-based or isolated strength criteria but should incorporate a multidimensional evaluation that includes neuromuscular symmetry and psychological readiness to support a safe and sustainable return to sport.

## Figures and Tables

**Table 1:** Comparison of pre/post-surgery limb symmetry indices (LSI, means ± SD) between quadriceps (Q) and hamstring (H) peak torque (PT) isokinetic values at 90°/sec and 180°/sec angular velocities. P = pre/post levels of significance; NS = not significant.

LSI	PT 90		PT 180	
	H	Q	H	Q
PRE	92.5 ± 12.7	100.5 ± 16.4	95.9 ± 12.3	100.5 ± 11.8
POST	87.5 ± 11.8	98.9 ± 10.2	92.6 ± 9.5	100.8 ± 11.8
P	0.015	NS	0.038	NS

**Table 2:** Comparison of pre/post-surgery limb symmetry indices (LSI, means ± SD) between athletes with and without ACL re-injury. Mann–Whitney U test was used to compare post-operative quadriceps (Q) and hamstring (H) limb symmetry indices (LSI) assessed at 90°/s and 180°/s between athletes who sustained a second ACL injury and those without re-injury during follow-up. Results are reported as Mann–Whitney U statistic (U), standardized Z value (Z), two-tailed *p*-value (*p*), and effect size (r).

	U	Z	*p*	r
Q LSI at 90°/s	239	−0.22	0.83	0.03
H LSI at 90°/s	214	−0.71	0.48	0.09
Q LSI at 180°/s	228	−0.44	0.66	0.06
H LSI at 180°/s	241	−0.18	0.86	0.02

## Data Availability

The data presented in this study are available from the corresponding author upon reasonable request.

## Supplementary Materials

The following supporting information can be downloaded at: https://www.mdpi.com/article/10.3390/jcm15031243/s1, Table S1: Isokinetic strength data, Peak Torque and Limb Symmetry Index at 90°/s and 180°/s for both operated and non-operated limbs, including Pearson’s correlation and Mann-Whitney results; Table S2: Demographic data of the participants (age, weight, height), return to sport status, incidence of new ACL injuries (re-injury), and Tampa Scale for Kinesiophobia (TSK) scores.
